# Management of overweight and obesity in children and adolescents by nurses: a mixed-method study*

**DOI:** 10.1590/1518-8345.6294.3789

**Published:** 2022-11-07

**Authors:** Renata Cardoso Oliveira, Rafaella Queiroga Souto, José Luís Guedes dos Santos, Altamira Pereira da Silva Reichert, Elisabeth Luisa Rodrigues Ramalho, Neusa Collet

**Affiliations:** 1 Universidade Federal da Paraíba, João Pessoa, PB, Brazil.; 3 Universidade Federal de Santa Catarina, Florianópolis, SC, Brazil.

**Keywords:** Child, Adolescent, Obesity, Overweight, Pediatric Nursing, Primary Health Care

## Abstract

**Objective:**

to analyze the management of overweight and obesity in children and adolescents by nurses of the Family Health Strategy.

**Method:**

this is a study of convergent parallel mixed methods, developed in Health Centers of a municipality in northeastern Brazil. In the quantitative stage, data were collected from a questionnaire applied to 98 nurses and analyzed by descriptive statistics. For the qualitative stage, semi-structured interviews were conducted with seven nurses, interpreted by inductive thematic analysis. The quantitative and qualitative results were integrated and presented by a joint display.

**Results:**

most nurses rarely checked waist circumference (77.6%), dyslipidemia (55.7%), blood glucose (42.3%), and neither evaluated blood pressure (75.3%). In the qualitative results, we identified that there are nurses who did not classify body mass index according to sex and age. As for medical tests, the requests were mainly related to the routine of childcare. Guidance on physical activity and diet were given in a basic way or attributed to other professionals, and referrals to other services or professionals were not followed up.

**Conclusion:**

it is imperative to train nurses for the management of overweight and obesity in primary care for children and adolescents, with a view to quality of care for the prevention of comorbidities.

## Introduction

Obesity has become a serious public health issue worldwide, considering that it increasingly reaches early age groups, has an epidemic character, and is considered a risk factor for other diseases^1^. The World Health Organization (WHO) defines obesity as a chronic multifactorial condition characterized by excessive accumulation of body fat, causing damages to health^2^.

In Brazil, overweight and obesity in children and adolescents is classified according to the WHO growth curves, available in the children’s and adolescents’ handbooks. The cutoff points for this classification of nutritional status are in accordance with the score or percentile. Children up to five years of age are considered overweight when the score is > + 2 and ≤ + 3 or > 97^th^ percentile and ≤ 99.9^th^ percentile and obese when the score is > + 3 or > 99.9^th^ percentile. In the age group from 5 to 20 years, when the score is > + 2 and ≤ + 3 or > 97^th^ percentile it is considered obesity, and when the score is > + 3 or > 99.9^th^ percentile it is defined as severe obesity^3^.

Obesity, in addition to increasing the risks for the clinical involvement of type 2 diabetes, cardiovascular diseases, certain types of tumors^4^ ^-^ ^5^, and the worsening of cases of COVID-19^6^, in children and adolescents it may cause respiratory difficulties, increased risk of osteoarticular diseases, insulin resistance, metabolic syndrome, dyslipidemias, hypertension, and psychological effects such as low self-esteem, social isolation, and eating disorders^3^ ^-^ ^5^.

Worldwide, about 40 million children and adolescents aged 5 to 19 years are overweight or obese^3^. The WHO estimates that the number of obese children on the planet will reach 75 million by 2025^7^. In Brazil, it is estimated that about 6.2 million children under 10 years of age are overweight and 2.9 million are obese. As for adolescents, these values reach 9.7 and 3.4 million, respectively^3^.

Regarding children assisted in Primary Health Care (PHC) services of the Brazilian Unified Health System (SUS), 14.8% of children under five years of age and 28.1% of children between five and nine years of age are overweight, and 7% and 13.2% of them, respectively, are obese according to the Body Mass Index (BMI) for age. As for adolescents assisted in PHC, 27.9% are overweight and 9.7% are obese^3^.

Although biological factors have a strong influence on obesity and overweight, the increase in their prevalence in recent decades is related to the adherence to unhealthy lifestyle habits^8^. Thus, obesity can be avoided or minimized if there is prevention and/or timely assistance focusing on recommendations aimed at children, young people, and their families to support a healthy diet to achieve good results, with consumption of more natural and less industrialized foods, physical activity, quality sleep, and reduction of the use of screens and sedentary lifestyle^9^.

Considering the complications caused by this morbidity, the high prevalence rates and the need to manage this chronic disease, managers and health professionals should be attentive and trained to minimize the incidence, prevalence, and consequences of obesity^10^.

The management of overweight and obesity in children and adolescents is one of the nurse’s attributions, a function anchored on a legal basis that legitimizes it, including actions related to anthropometry, assessment of nutritional status, guidance on a healthy lifestyle, request for tests, identification of risk factors and more recurrent morbidities associated with overweight, and referrals to other professionals when necessary^3^ ^,^ ^11^ ^-^ ^14^.

Although studies on the role of PHC nurses in the management of overweight and obesity in children and adolescents are still considered scarce, recent publications show weaknesses in the professionals’ conduct to implement the promotion, prevention, and treatment of this morbidity^14^ ^-^ ^19^. Among them are studies conducted in Australia^17^, Sweden^19^, and the United States of America^20^. Identifying this management is the first step to support the development of specific protocols and training for this professional category, aiming at the search for improvements in the provided care as well as better quality of life for children and adolescents.

Taking this into consideration, we aim to analyze the management of overweight and obesity in children and adolescents by nurses of the Family Health Strategy. 

## Method

### Study design

This is a study of mixed methods, of the convergent parallel type, which is characterized by the collection and analysis of qualitative and quantitative data simultaneously and independently. At the end of the study, the results are integrated in search of convergences and/or divergences between them, considering the same weight allocation to the two approaches (QUAN + QUAL)^21^.

In this sense, a cross-sectional study was developed in the quantitative approach and an exploratory-descriptive research for the qualitative approach. The use of the mixed method is justified by the possibility of a deeper and more detailed understanding of the object of study, by the integration of quantitative and qualitative approaches. Hence, this methodology allows investigating both actions for the management of overweight and obesity developed by nurses and the way they are performed.

Concerning the observance of the methodological rigor of the study, the instrument Strengthening the Reporting of Observational Studies in Epidemiology (STROBE) was used for the quantitative element; and the Consolidated Criteria for Reporting Qualitative Research (COREQ), for the qualitative element. In addition to these guides of the EQUATOR Network, the recommendations of the Mixed Methods Appraisal Tool (MMAT)^22^ ^)^ were adopted for the methodological rigor of mixed studies.

### Data collection site

Data was collected at a UBS in the municipality of Campina Grande, state of Paraíba (PB), Brazil.

In the aforementioned city, the Primary Health Care network, in 2019 (time of data collection), had a FHS coverage ranging from 88% to 89.6%, with 87 UBS in rural, urban, and surrounding districts under the responsibility of the city. These UBS in the Department of Health of this municipality were grouped in the territory into Health Districts (HD), with the following distribution of teams: 15 Family Health teams (FHT) in HD I; 12 FHT in HD II (three of which in rural areas); 15 FHT in HD III; eight FHT in HD IV (three of which in rural areas); 11 FHT in HD V; 12 FHT in HD VI (three of which in rural areas); six FHT in HD VII; three FHT in HD VIII; 13 FHT in HD IX; and 11 FHT in HD X. Therefore, in total, there were 106 FHT, with one nurse in each team.

### Study period

Data were collected between May 2019 and March 2020.

### Study population

The study population consisted of 106 nurses from the FHT that worked in the urban, rural, and district UBS, under the responsibility of the Municipal Department of Health of the study municipality.

### Selection criteria

The inclusion criteria were: to work in the FHT of UBS in the urban, rural areas, and administrative districts that were under the responsibility of the Municipal Department of Health of Campina Grande (PB); and perform their function for a period of more than three months. The exclusion criteria were: being on vacation, on medical leave, or leave of absence; being an intern or substitute nurse of the UBS. The authors deemed loss after the fourth unsuccessful attempt to meet the professional for data collection.

### Data collection

Data from both approaches were concomitantly collected. The qualitative interview was conducted before the application of the quantitative instrument, in such a way that its structured items did not influence the participants’ answers. Data were collected in the period prior to the new coronavirus pandemic; therefore, the interviews were conducted in person at the UBS and took place when the nurses finished their work shifts or when they had a free moment between appointments.

### Quantitative element

#### Participants

The quantitative approach was performed by a census^23^, that is, with the population of nurses from the FHT of the municipality under study, except for losses and exclusions. After applying the inclusion and exclusion criteria, of the 106 nurses registered in the Department of Health of the municipality, seven professionals were excluded, one for having less than three months of work at the institution, three for being on vacation, and three for being on bonus leave. There was also a loss after the fourth contact attempt. Therefore, 98 professionals participated in the quantitative approach.

#### Study variables

Quantitative data were collected by a questionnaire prepared based on the guidelines and manuals of the Brazilian Ministry of Health (MH) for the management of overweight and obesity in children and adolescents, containing 25 questions with nominal and ordinal categorical variables, which included the following aspects: materials available at the UBS for the management of overweight, obesity, and its comorbidities; anthropometry, which consisted in the evaluation of anthropometric measurements of children and adolescents performed by nurses; anamnesis, which verified the actions performed by nurses in search of the family and personal history of these young people related to this morbidity as well as guidelines for the adoption of a healthy lifestyle; morbidities associated with overweight, which evaluated what nurses did to identify the consequences of obesity; and the School Health Program (*Programa Saúde na Escola -* PSE), which investigated how health care is provided by these professionals in the school environment.

#### Instruments used to collect information

A structured instrument was prepared, considering that no questionnaire was found in the literature to assess the management of FHS nurses concerning overweight and obesity in children and adolescents. This instrument was developed focusing on the guidelines for the management of overweight and obesity contained in the handbook *Strategies for the care of the person with chronic disease: Obesity* ^11^, of the Brazilian Ministry of Health, and in the Manual of the Brazilian Society of Pediatrics for children and adolescents with obesity^12^. It was also based on Law No. 7498/1986, concerning the professional practice^24^, Resolution No. 195/1997, which provides for the request for routine and complementary tests by nurses^25^, and the Nurse’s Protocol in the FHS of the State of Paraíba^13^.

To evaluate the reliability and internal consistency of the data collection instrument, the Cronbach’s alpha coefficient was applied, whose results vary between 0 and 1, with no negative limits. The closer the Cronbach’s alpha coefficient is to 1, the greater the internal consistency of the scale items. Thus, the parameters for evaluation are as follows: α>0.9 - excellent; α>0.8 - good; α>0.7 - acceptable; α>0.6 - questionable; α>0.5 - bad; and α<0.5 - unacceptable^26^. In this sense, the domain “UBS Materials and Anthropometry,” although it did not reach the reliability value of 0.7, was maintained in the analysis due to its importance for the evaluation and management of overweight and obesity. Moreover, the Cronbach’s alpha value obtained in this dimension is not considered bad or unacceptable. Therefore, the items are homogeneous and the instrument adequately evaluates the construct to which it was applied (Table 1).

**Table 1:** Cronbach’s alpha values related to the dimensions of overweight and obesity management of children and adolescents performed by nurses of the Family Health Strategy. Campina Grande, PB, Brazil, 2019-2020

Nurses’ actions	Cronbach’s alpha
**UBS Materials and Anthropometry**	0.605
**Anamnesis**	0.742
**Morbidities**	0.747
PSE	0.730
**Total**	0.706

### Data collection

The quantitative questionnaire was applied face-to-face with the participants, and the questions were impartially asked to the nurses.

#### Data processing and analysis

The Statistical Package for the Social Sciences (SPSS) software, version 18.0, was used for descriptive analysis of all variables by absolute and relative frequencies, measures of central tendency (mean) and dispersion (standard deviation). Missing data were processed as “missing.”

### Qualitative element

#### Participants

Seven nurses from different HD were interviewed, selected by convenience sampling, considering the availability of time to participate in the interviews, which were conducted in the nurses’ work environment.

#### Instruments used to collect information

An interview was conducted with a semi-structured script, based on the following guiding question: how is the management of overweight or obesity carried out with children and adolescents?

#### Data collection

The interviews were conducted in a private room available at the UBS and recorded in audio by digital media, with an average duration of 43 minutes each, after the interviewees’ consent. The participants were led by the main researcher of the study, a doctorate student. Regarding the reliability of the qualitative approach, the precepts contemplated by Sandelowski^27^ ^)^ were followed. Field notes were made after the interviews.

The end of the collection occurred according to the criterion of sufficiency, when the authors certified that an internal logic of the data was achieved, enabling to establish a comprehensive framework of the object of study^28^.

#### Data processing and analysis

The interviews were fully transcribed and interpreted by inductive thematic analysis (ITA)^29^. The conceptual framework that delimited this analysis was the management of overweight and obesity in children and adolescents contained in the national guidelines^3^ ^,^ ^11^ ^-^ ^14^ with regard to Laws, Resolutions, and the Nurse’s Protocol^24^ ^-^ ^25^.

ITA was developed in six steps. In the first, data were familiarized by transcription, reading, and rereading to survey initial ideas. In the second, the initial codes were produced from the systematized organization of the data set in significant semantic groups. In the third, the search for potential topics was performed by grouping the codes. In the fourth step, the formulated topics were reviewed to confirm whether they were in accordance with the coded extracts and the data set. In the fifth, a new refinement analysis was carried out to name the topics. In the sixth and last step, the report that is presented in the results section of this study was prepared.

Although it is a sequential process, it is worth emphasizing that these steps are flexible and allowed a back-and-forth movement through the data set, coded extracts, and the analysis performed throughout the steps^29^. Based on the groupings, 65 codes were identified, which generated the topic: “Management of overweight or obesity in children and adolescents performed by nurses of the Family Health Strategy (FHS).”

After quantitative and qualitative analyses of the data, independently and following all the specific methodological rigor for each of the elements, the data were integrated. In the integration, the quantitative and qualitative results were compared to identify convergences and divergences, as well as combinations for better understanding in the response to the general purpose of the study, interpreted in a single conclusion^21^.

The integrated data are presented in a joint display in the Results section.

### Ethical aspects

This study was developed in accordance with Resolution No. 466/2012 of the National Health Council (CNS) of the Brazilian Ministry of Health and its supplementary resolutions, having been approved by the Research Ethics Committee under Opinion No. 4.174.864 and Certificate of Presentation for Ethical Consideration (CAAE) 10627619.9.0000.5188. All participants signed an Informed Consent Form and were identified by “N” followed by the numeral corresponding to the increasing order of the interviews to guarantee anonymity.

## Results

Nurses were predominantly women (n=94; 95.9%), with a mean age of 43.46 years (standard deviation [SD])=9.24), with a minimum of 25 and a maximum of 65 years. Regarding academic degree, 83.7% had a specialization degree (n=82), 15.3% had a Master’s degree (n=15), and 1% (n=1) had a PhD degree.

The quantitative results showed that most nurses requested tests (n=53; 54.6%). However, 55.7% did not regularly request tests to assess dyslipidemia and 42.3% to assess blood glucose in children and adolescents diagnosed with overweight. In addition, most nurses rarely checked the waist circumference (n=77; 77.6%) and blood pressure (n=73; 75.3%) of these individuals, and 59.8% reported only having the blood pressure monitor for adults in their UBS (Table 2).

**Table 2:** Evaluation of cardiovascular risks of children and adolescents diagnosed with overweight by nurses of the Family Health Strategy (n=97). Campina Grande, PB, Brazil, 2019-2020

Characteristic	Never	Rarely	Sometimes	Always	Total
**Dyslipidemia**	28 (28.9)	11 (11.3)	15 (15.5)	43 (44.3)	97 (100.0)
**Blood glucose**	14 (14.4)	9 (9.3)	18 (18.6)	56 (57.7)	97 (100.0)
**Evaluation of waist circumference**	48 (49.0)	9 (9.2)	19 (19.4)	22 (22.4)	98 (100.0)
**Blood pressure**	52 (53.6)	9 (9.3)	12 (12.4)	24 (24.7)	97 (100.0)

Most professionals did not use the form of the Food and Nutrition Surveillance System (SISVAN), but provided guidance on healthy eating (n=83; 85.6%), physical activity (n=57; 58.2%), and referred patients to other professionals or another healthcare sector (86.6%; n=84) (Table 3).

**Table 3:** Actions performed in the appointment of children and adolescents diagnosed with overweight by nurses of the Family Health Strategy (n=98). Campina Grande, PB, Brazil, 2019-2020

Characteristic	Yes	No	Total
	n (%)	n (%)	n (%)
**Food assessment (Sisvan form)**	26 (26.5)	72 (73.5)	98 (100.0)
**Guidance on eating practices**	83 (85.6)	14 (14.4)	97 (100.0)
**Guidance on physical activity**	57 (58.2)	41 (41.8)	98 (100.0)
**Referral to other professionals or other healthcare sector**	84 (86.6)	13 (13.4)	97 (100.0)

By the inductive thematic analysis of the qualitative data set, we developed the topic “Management of overweight or obesity in children and adolescents performed by nurses of the Family Health Strategy (FHS)”. The nurses presented weaknesses in knowledge and practice to manage overweight or obesity in children and adolescents. We evidenced that the professionals did not check or evaluate waist circumference: *The* [waist] *circumference, we only check it in hypertensive* [adults]. *And if it’s actually an obese teenager or child, I don’t know if the doctor requests it, I don’t* [request it]*.* […] *Usually it’s just weight and height* (N7).

The participants explained some parameters for blood pressure assessment in the juvenile population: *No, an adult over 14/9 is already altered. But a child over 12/8, 13/9, approximately, is already alarming as well* (N4).

Some nurses only evaluated BMI, without considering the handbook chart according to age and sex. *By the time I open the PEC* [*Prontuário Eletrônico do Cidadão -* Electronic Citizen’s Record]*, as she* [nurse technician] *has already registered the measures, it* [BMI] *is already there for me as it is. Then, nowadays, I use the chart less and work more with just BMI, really* (N7).

Regarding the request for tests, they were limited to those of the routine of childcare, which are not suitable for the evaluation of possible complications related to overweight. *The* [tests] *of childcare itself: complete blood count, cholesterol, triglycerides, hum… blood glucose, urinalysis and parasitological stool sample, which is the protocol of childcare* (N2).

The limitations of the National Regulation System (SISREG) were also pointed out as justification for not requesting tests from overweight children. *Doctors are the ones who request tests; as we know that there’s a regulatory system today, there are some criteria, sometimes I’m afraid to request some tests and, when you insert it in the system, be prevented because it was requested by the nurse* (N6).

The nurses believed that guidance on physical activity was the responsibility of the physical educator. *The physical educator should do this* [provide guidance about physical activity]*, the nurse doesn’t do this* (laughs). *We don’t have this type of training and guidance in primary health care* (N2).

When identifying overweight in a child, some nurses made basic feeding recommendations and referred them to the nutritionist. However, others referred these children without nutritional counseling. *I refer children who are diagnosed with obesity to the nutritionist.* […] *I leave the dietary plan to the nutritionist, because it’s not my job* [to do it] (N3).

When they referred children to the nutritionist, overall, they did not follow the itinerary and therapeutic outcome of the overweight child or adolescent, nor was there dialogue between the nurse and the professionals who received the referral. *After I referred* [patients] *to her* [nutritionist], *there’s no coming back* […] *only if it’s a child undergoing childcare treatment, then* [the child] *will really come back later for subsequent appointments* (N3).

**Figure 1:**
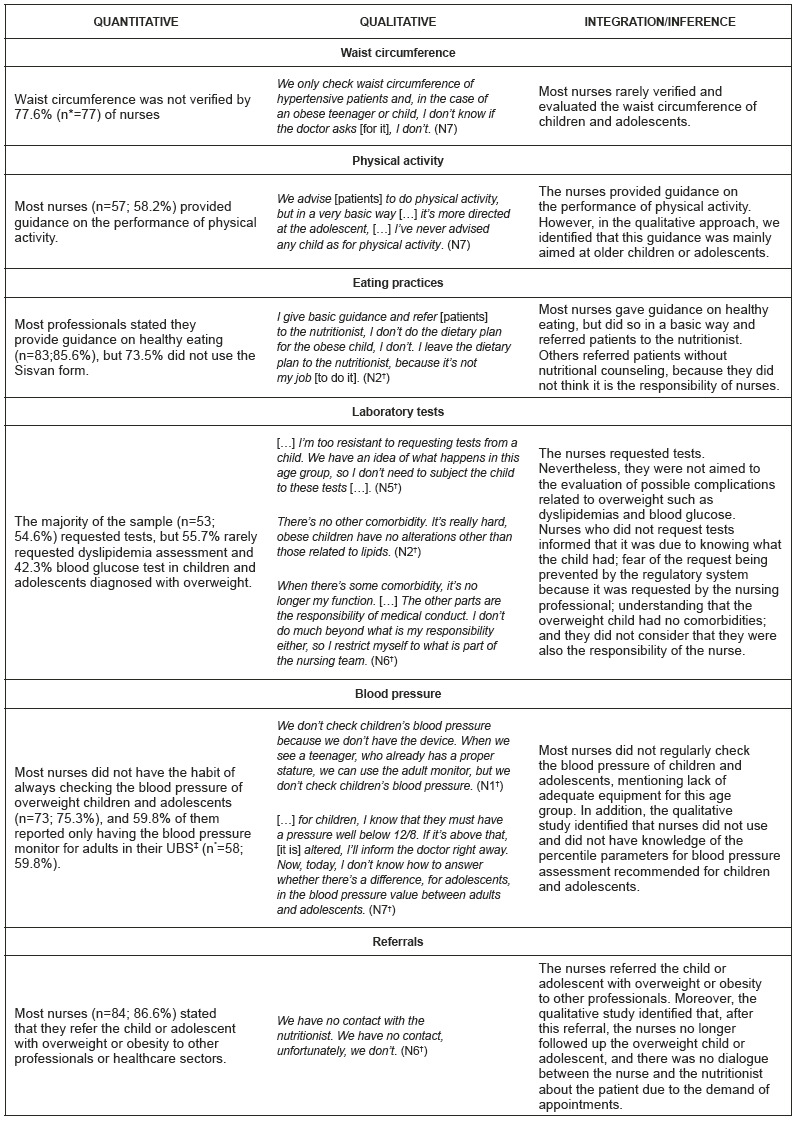
Concomitant integration of data on the management of overweight/obesity in children/adolescents by nurses from the Family Health Strategy (n=98). Campina Grande, PB, Brazil, 2019-2020 *n=number; ^†^N=nurse; ^‡^UBS=Health Center.

The results of the integration of quantitative and qualitative data are presented in a joint display, which simultaneously displays the quantitative and qualitative results and their integration, enabling to better visualize the information. There was convergence in all elements. In the qualitative element, we could also identify singularities (Figure 1).

## Discussion

The analysis of the management of overweight and obesity in children and adolescents performed by nurses of the FHS showed weaknesses in the knowledge and practice of these professionals.

The consequences of obesity affect the entire stage of growth and development of children and adolescents and may remain in the short, medium, or long term. This condition is associated with a higher chance of premature death, maintenance of obesity, and disability in adulthood^3^.

The literature recommends identifying and tackling this morbidity at younger ages and provide guidance directed to these individuals and their families on the adherence to a healthier lifestyle^11^ ^,^ ^30^.

In order to perform this management, according to the handbook *Strategies for the care of the person with chronic disease: Obesity, of the Brazilian Ministry of Health* ^11^, and the Handbook for the care of overweight and obese children and adolescents at Primary Health Care^3^, nurses of the FHS can perform anthropometry; assessment of nutritional status; approach and stimulus to the adoption of a healthy lifestyle; request for tests of blood glucose, cholesterol, triglycerides, blood glucose and insulin curves, glutamic oxaloacetic transaminase (GOT), glutamic pyruvic transaminase (GPT), gamma-glutamyl transferase (GGT), thyroid-stimulating hormone (TSH), and thyroxine (Free T4), which identify the most recurrent comorbidities. In addition, they can refer young people to other professionals and/or services when necessary. All these actions comply with Ordinance No. 2.436/2017, which approves the National Policy on Primary Health Care^31^; Law No. 7498/1986^24^, the regulations of the Federal Nursing Council (Cofen); Resolution No. 195/1997, which provides for the request for routine and complementary tests by nurses^25^; and the Nurse’s Protocol in the FHS of the State of Paraíba^13^.

Regarding the work process in primary health care for the management of overweight, the electronic medical record has been an important tool, as it provides the calculation of BMI after insertion of the child’s weight and height. However, using this datum alone, without considering the appropriate evaluation parameters for sex and age^19^ ^-^ ^20^, can lead to unreliable interpretations.

The WHO recommends the use of growth curves to measure, monitor, and evaluate children and adolescents from zero to 19 years of age to detect juvenile overweight and obesity^3^.

Waist circumference (WC) is an important datum for the evaluation of overweight and obesity in children and adolescents^11^ ^-^ ^12^. Nevertheless, we evidenced that nurses of the FHS participating in the study did not verify nor evaluate this measure. This is worrisome, because WC, in addition to being a fast and low-cost measure, is strongly associated with high blood pressure in children and adolescents, even more than BMI itself, which is routinely evaluated by nurses^32^.

A study conducted in Spain on 265 schoolchildren aged from 6 to 17 years identified that, in early ages, obesity has been directly related to the development of high blood pressure (BP), with waist circumference being the measure that presented the highest association with hypertension (odds ratio [OR]=10.7), when compared with BMI (OR=7.5), waist-to-height ratio (OR=5.5), and body fat percentage (OR=5.3) (p<0.05)^32^. Considering that hypertension is a precursor of heart, cerebrovascular, and metabolic diseases^32^, the importance of measuring WC is emphasized.

The data demonstrated that the lack of adequate material affects the non-verification of BP in children and adolescents in PHC. However, according to the recommendations of the Brazilian Society of Pediatrics and the Brazilian Society of Cardiology^12^, BP measurement should be performed in any clinical evaluation from the age of three, following different parameters of the adult examination. Age, sex, and height percentile should be considered for BP assessment in children and adolescents^32^.

Overweight or obese children may develop morbidities associated with overweight^34^. Thus, the request for adequate tests is an important care in the management of this health problem. A study conducted in the United States of America also identified that most nurses do not investigate diseases associated with obesity, and some of them do not require specific blood tests for obese children^35^.

In Brazil, Law No. 7.498/1996 provides for the prescription of BP test in the nursing appointment, while Cofen Resolution No. 195/1997^25^ provides for the request for routine and complementary tests by nurses.

The Nurse’s Protocol of the FHS of the state in which this study was conducted allows nursing professionals to request the following tests for children: complete blood count; fasting glucose; glycated hemoglobin; total cholesterol and fractions; triglycerides; oral glucose tolerance test (OGTT), triiodothyronine (T3), thyroxine (T4), and thyroid-stimulating hormone (TSH)^13^.

For adolescents, in addition to the aforementioned tests, glutamic oxaloacetic transaminase (GOT) and glutamic pyruvic transaminase (GPT) are allowed^13^. All of them investigate comorbidities associated with obesity, which are risk factors for cardiovascular and metabolic diseases.

Resolution No. 195/1997 provides for the request for routine and complementary tests by nurses and emphasizes that if these professionals do not request tests from patients, when necessary, their act is considered omission, negligence, and recklessness^25^. Therefore, it is essential to understand that obesity is a chronic disease and requires multiprofessional care. Furthermore, it is paramount that the SUS Sisreg be aware of this permission and perform the tests requested by PHC nurses.

The adoption of these measures may result in a reduction in waiting lines for medical appointments by young people and their family members. A study conducted in Canada states that actions carried out directly by nurses to children and adolescents with obesity reduce waiting times, as they do not need to go through the doctor^36^. Moreover, from these strategies, there may be a strengthening of PHC as a coordinator of health care for secondary and tertiary care of the health system.

In addition to anthropometric data, blood pressure measurement and request for tests, guidance, and referrals are part of the follow-up protocol of these children^12^. Only identifying obesity and registering it in the handbook of children and/or adolescents are not sufficient actions to reduce the prevalence of this health issue^10^.

To improve this practice on healthy eating, the MS recommends the use of the Dietary Guidelines for the Brazilian population, which presents all the guidelines on eating for different age groups, including children and adolescents^37^.

Identifying the problem of eating practices, providing nutritional advice, and/or referring for evaluation with a nutritionist is an important care, but not sufficient. Besides, it is essential to follow up the itinerary and therapeutic outcome of the overweight child or adolescent, in addition to establishing dialogue with the professionals who received the referral to propose a singular therapeutic project with follow-up of the team.

Parents of overweight or obese children in Australia reported feelings of lack of problem-solving capacity after nurses identified their children’s excess weight. They claim not to have received enough information about healthy eating practices by professionals^17^. Other studies have identified that nurses, when referring overweight children or adolescents to the nutritionist, do not have a dialogue with this professional^19^ ^,^ ^36^ as well as no longer follow up these young people after referral^20^ ^,^ ^38^. These results corroborate the findings of our study.

To solve this problem of dialogue between parents and professionals, nurses in Canada who were referring obese children and adolescents to a reference center in the treatment of this morbidity requested that a counter-referral be sent with updates, informing about the situation of these young people and their families. Thus, they contributed to a better communication between professionals and to the continuity of care to this group^36^.

In addition to the guidelines on healthy eating and referrals to the nutritionist, it is important to value the performance of physical activity. In this study, as in another research^17^, the guidance provided by nurses were basic and focused on older children. However, for a healthy life, physical activity must be stimulated and performed from the first years and continue throughout life^12^ ^,^ ^39^.

Thus, children from zero to two years should be encouraged to be as active as possible, in a safe, supervised, and stimulating environment. Children under one year of age should do at least 30 minutes per day of physical activity face down; those aged one to two years, at least three hours of any intensity; those aged three to five years, at least three hours per day, with at least one hour of moderate to vigorous intensity. For those older than six years of age and adolescents, 60 minutes or more of physical activity is recommended. These activities can be distributed throughout the day^39^.

A study identified that nurses do not guide parents about physical activity, due to the lack of confidence in their knowledge^17^ ^)^ and agree on the need for training on the subject^19^ ^-^ ^20^. In the present study, in addition to the identification of nurses’ limited knowledge of physical activity, we noticed that they do not provide these guidelines because they believe in a conception of division of tasks between professional categories, and that the guidance on this practice are the sole responsibility of the physical educator.

It is of fundamental importance for nurses to provide these guidance in their appointments, as these young people, when having a multiprofessional care, have better compliance with treatment recommendations and more effective results^19^ ^-^ ^20^. It is also essential to resume that changes in behavior should be extended to the entire family, aiming to obtain greater treatment adherence and better outcomes of the patients^40^.

To improve the knowledge and practice of nurses, it is necessary to invest in continuing education on obesity and skills in its management as well as to reorganize the health system to follow up people with this morbidity^17^ ^,^ ^19^.

In addition, other initiatives may contribute to best practices in the management actions of juvenile overweight and obesity. Among them are the development of specific protocols for each professional healthcare category, including nursing, so that these groups can understand what are their conducts in the care of this morbidity; and the increase in human resources so that professionals have greater availability of time to perform this management.

Due to the use of the quantitative and qualitative approach, it was possible to minimize the weaknesses of both methods, because the positive points of one strategy compensated for the weaknesses of the other. As a limitation, we point out the nonuse of a quantitative research instrument validated for the evaluation of the management of overweight and obesity aimed to children and adolescents.

## Conclusion

When analyzing the management of overweight and obesity of children and adolescents by nurses of the FHS, weaknesses in knowledge and practice were evidenced. Specific protocols for FHS nurses and continuing health education are important strategies for better practices.

By performing adequate management of overweight and obesity of the juvenile population in PHC, it will be possible to provide opportunities for a better quality of life for these children and young people, as well as to stimulate the reduction of comorbidities in their lives as adults - such as cardiovascular and metabolic diseases, which have shown high mortality rates in increasingly young populations. Moreover, healthier adults reduce SUS costs, as chronic diseases require high investments for treatments and hospitalizations.
